# A mobile methods pilot study of surgical spaces: ‘**f**it for purpose? Organisationa**l** pr**o**d**u**ctivity and wo**r**kforce wellbe**i**ng in work**s**paces in **h**ospital’ (FLOURISH)

**DOI:** 10.1186/s12913-020-4938-8

**Published:** 2020-02-03

**Authors:** Frances Rapport, Emilie Francis-Auton, John Cartmill, Tayhla Ryder, Jeffrey Braithwaite, Robyn Clay-Williams

**Affiliations:** 1 Australian Institute of Health Innovation, Macquarie University, Sydney, Australia; 2 Macquarie University Hospital, Macquarie Park, Australia

**Keywords:** Mobile methods, Work-as-imagined, Work-as-done, Photographs, Workforce, Workspace

## Abstract

**Background:**

Good workspace design is key to the quality of work, safety, and wellbeing for workers, yet we lack vital knowledge about optimal hospital design to meet healthcare workforce needs. This study used novel mobile methods to examine the concept of Work-as-Done and the effect of workspace-use on healthcare professional practice, productivity, health and safety in an Australian university hospital.

**Methods:**

This pilot study took place in one gastroenterological surgical unit between 2018 and 2019. Data collection involved 50 h of observations and informal conversations, followed by interpretation of five architectural plans and 45 photographs. Fieldnotes were thematically analysed and corroborated by analysis of visual data using a predefined taxonomy.

**Results:**

Six themes were identified, revealing spaces that both support and hinder Work-as-Done. Fit-for-purpose spaces facilitated effective communication between staff, patients and families, conferred relative comfort and privacy, and supported effective teamwork. Unfit-for-purpose spaces were characterised by disruptions to work practices, disharmony among team members, and physical discomfort for staff. Staff employed workarounds to manage unfit-for-purpose spaces.

**Conclusion:**

The results identified negative impacts of negotiating unfit-for-purpose workspaces on the work and wellbeing of staff. While the use of workarounds and adaptations enable staff to maintain everyday working practices, they can also lead to unexpected consequences. Results indicated the need to identify and support fit-for-purpose spaces and minimize the detrimental qualities of unfit-for-purpose spaces. This study showed that mobile methods were suitable for examining Work-as-Done in a fast-moving, adaptive hospital setting.

## Background

### Workspace design

Workspaces are socially constructed environments and active parts of our lives [1]. They are places where “social relationships are produced, reproduced, contested and transformed” [2]. The primary function of workspace is the delivery of a service or product of the highest quality [3, 4] to the benefit of, in the case of healthcare, patients. Workspaces should also enable employees to feel safe and healthy [3, 4]. When these primary factors align, secondary benefits result, including physical and psycho-social wellbeing [5–7], productivity [8], and economic viability [9–11], and when managed effectively, workspaces are then said to be ‘resilient’ [3].

Studies of workspace design, across different industries, have predominantly concentrated on ergonomics [12, 13], stress and safety in the workplace [14]. Studies have assessed the effects of stress on individual and organisational productivity and job satisfaction and found clear correlations with absenteeism and lower job satisfaction [15, 16]. This has led to new socio-behavioural interventions such as healthy eating and fitness regimes [14, 17]. Globally, it is recognised that crowded hospitals and the lack of individualised care exacerbate staff stress, while good workspace design is key to the quality of people’s working lives (happiness, health and productivity) [18]. Yet worldwide, healthcare employees, vital players in national safety, are frequently not engaged in hospital planning [19] and in new hospitals in particular, rarely asked about their needs or the needs of their patients.

### Work-as-imagined and work-as-done

Hospitals are currently designed according to the Work-as-Imagined (WAI) model [20]. This is based more on a top-down, managerial imperative relating to how work is performed than to an in-depth understanding of what actually happens in the workplace. Work-as-Done (WAD), on the other hand, not only represents what is actually taking place, but recognises that continual adjustments to systems and processes are needed if employees are to function effectively [20]. Adjustments reflect not only how systems evolve and flex, but also how working in unsuitable spaces can lead to poor outcomes including unsafe practices and demoralisation. Until hospitals are adequately designed to match the needs of the workforce, expensive, impactful mistakes are likely to continue, creating a lowering of standards and a pressurized workforce [18, 21].

### Working hours and unsafe practices

In 2015 in Australian healthcare (where this study took place), over 460,000 registered healthcare professionals (HCPs) were recorded as working a 42–72 h week (rather than the prescribed 38 h) with one in two doctors working unsafe hours [22, 23]. This has been associated with tiredness, inefficiencies in the workplace, and ill-placed technology and objects in space [23, 24], adding to a growing number of unsafe practices, such as the removal of the wrong body parts during operations or foreign bodies remaining in patients after operations.

Currently, one-in-ten patient encounters in Australia is associated with a medical error which has been extrapolated as leading to 18,000 people dying unnecessarily each year [25]. Errors are also estimated as the third leading cause of death in the United States [26], while their reduction in the European Union, it has been calculated, would prevent “more than 750 000 harm-inflicting medical problems per year … and 95 000 fewer deaths” [27].

An increase in error reporting has created a whistleblowing society and de-motivated healthcare workforces with growing psychosocial problems (including burnout and suicide) now characterised as at ‘epidemic’ proportions [26, 28, 29]. Add to this an ageing global population, hospitalisation rates growing yearly [30] and hospitals stretched to capacity [31], and a critical re-think is needed, on a global scale, to design resilient healthcare environments based on WAD.

## Aims

1. Assess WAD in terms of surgical gastroenterological (GI) workspaces and their influence on workers’ productivity, health and safety;
2. Examine the impact of mobile methods-use on data collection in hospital surgical spaces;
3. Describe and visually illustrate professionals’ and patients’ personal experiences of a surgery unit’s working environment.

## Objectives

1. Clarify how a single GI surgery unit (the ‘case’) arrange, traverse and share workspace with others;
2. Reveal how different case members manage work in relation to spatiality and objects and spaces within and beyond the surgical theatre;
3. Disclose individual and team members’ views of WAD (including enabling and disabling qualities), productivity, health and safety, and wellbeing;
4. Present the complexities of the case, including the impact of workspace-use on people’s lives.

Through the translation of findings into practical outcomes, the study team aims to inform the design of Australian hospitals in keeping with workforce needs, and consider how environments could be safer, healthier and more productive places for people to work in.

## Methods

### Study design

A qualitative, intra-method pilot study used: (1) in-situ observations of spatial use, (2) shadowing and informal conversations with HCPs and patients (‘mobile methods’) [32, 33] and (3) the collection of images (drawing, photographs and architectural plans) of the spatial layout of one private hospital’s GI surgery unit. The study was conducted between June 2018 and April 2019.

### Conceptual framework

In complex adaptive systems such as healthcare, WAI, a concept from the resilient health care field [21], drives the design of the majority of workspaces. WAI reflects how work is understood by those who are separated from it by time or space [34] (e.g. architects and policymakers). WAI results from an over-simplified version of what is actually going on, including the small and large adjustments HCPs make daily to manage their workloads. WAD, on the other hand, is defined by people’s actual roles and responsibilities, and takes account of what it means to function effectively, despite fragmented, complex, and resource-restrained circumstances [3, 20]. Understanding WAD requires a realistic exploration of working practices and perceptions, as well as an understanding of systems and settings, approaches to policy, and aspirations for improvement. In this study we examined WAD, taking into account Fox’s [35] three ‘circuits of hygiene’ in operating theatres: 1) the instrument circuit (route to clean and sterile equipment separated from contaminated equipment), 2) the staff circuit (staff-only access points regulating entry and exit to theatres), and 3) the patient circuit (access to patient holding rooms and the anaesthetic room). In addition, using mobile methods, we moved beyond the body of research defining such circuits and social relationships, to examine a broader suite of issues relating to workforce health, wellbeing and productively, and professional perceptions of workspace-use and safety [35–37].

### Methodology: Mobile methods

Studies on workspace in Australia and internationally have overwhelmingly focused on client or manager perspectives rather than employee experience [38], prioritising self-reporting (questionnaires and focus groups) over observations from researchers’ immersion in the landscape, failing to consider functionality in time and space, or WAD from worker perspectives. Mobile methods can help with this, capturing the increasing mobility of people, goods and objects, while enabling descriptions of organisational structure, adaptation and complexity, and through images, ideas and texts, people’s sense of identity, interaction, and power [39].

This pilot study used mobile methods ‘on the hoof’ (rarely attempted in health services research) [40]. Methods included a dedicated study researcher’s observations, fieldnotes, and informal conversations as she moved alongside participants going about their daily work [32]. Focusing on a narrow aspect of mobile methods called ‘shadowing’, the study researcher (EF-A) followed “selected people in their everyday occupations for a time” [41], to: 1) examine patterns of social, occupational and spatial engagement and people’s experiences of what was happening around them [42], 2) consider the complexity of the GI surgical site within and beyond the operating theatre, 3) assess how patients were managed and results were achieved, 4) question the meaning of WAD, 5) reflect on professional and social change [41], and, 6) gain insight into roles, responsibilities and hierarchies.

### Setting and sample

The case site was a private teaching hospital located within an Australian university campus, co-located with a medical Faculty, comprising 182 beds, 12 operating theatres, 2 cardiac and angiogram suites and other facilities accessed by the surgery unit. The hospital (opened in 2010) is privately funded, and considered an affluent, metropolitan organisation, which is dedicated to a culture of continuous improvement through research and education. The hospital employs cutting-edge diagnostic and treatment technology and keeps electronic medical records, making it a good example of contemporary Australian hospital architecture.

There are notable differences between private and public hospitals in Australia worth noting in the context of assessment of hospital spaces and care services provided in those spaces, particularly regarding funding and the populations served. Private hospitals are generally smaller than public hospitals and while treatment in public hospitals does not incur out-of-pocket costs for the patient, as these services are funded by federal and state or territory governments, most care in private hospitals is funded through private health insurance and patient payments. Private hospitals also tend to treat patients with fewer comorbidities and focus on planned/elective surgery, while more complex patients are treated in public hospitals. Individuals living in regional/remote areas or disadvantaged communities are more likely to be treated as public patients than those living in major cities and in the least disadvantaged areas [43]. As such, public hospitals serve a much broader demographic and accounted for the majority of hospitalisations in Australia (and nearly all unplanned admissions) in 2017–2018 [44]. The different patient demographic served in private hospitals in Australia, and in general, the smaller hospital setting, with more individual patient rooms, closely resembles general global differences between private and public hospitals in developed or developing countries.

The site was contained and easily identifiable, lending itself to an in-depth observational investigation. It offered a setting where personal discussions took place and collaboration was crucial to staff and patient safety. Operating theatres and their linked spaces were one of the most inaccessible areas of the hospital, making it a challenging yet vital testbed for spatial investigation, and while use of space in operating theatres was highly regulated, theatres abutted more relaxed, open spaces, making it an appropriate site to examine people’s movements and actions.

The study focussed on one small, fixed-member team, caring for a culturally homogenous, predominately English-speaking population including a typical arrangement of two consultant surgeons, two scout nurses, one scrub nurse, one anaesthetist, and one anaesthetist nurse. Surgeons worked individually for relatively straight-forward procedures (such as colonoscopies or hernia repairs), and in pairs for more complex cases (such as low anterior resection for rectal cancer).

### Recruitment procedure

#### Group 1 HCPs

HCPs were recruited using purposive sampling [45] to ensure a varied cohort and working patterns. A study briefing meeting for the case team and wider hospital staff was followed by the signing of study consent forms. The briefing meeting included a description of the study and details about data capture methods. Staff were also informed that any interactions with members of the wider workforce would only be recorded if consent was obtained and that no identifiable data would be collected nor any clinical details about patients and families.

#### Group 2 patients

While patients were not the focus of the study, and thus no patient demographic data were collected on this occasion, it was recognised that incidental observations could take place, thus a responsible patient recruitment approach was designed using time-frame sampling [46]. This ensured each patient had an equal opportunity of being recruited over a predefined time period. Recruitment was undertaken in the order in which patients agreed to take part in the study and led to the first 20 patients due to undergo surgery, aged 18 years or older, being contacted in the order in which were put onto the case surgery list. A dedicated clinical liaison officer consented patients to remove team involvement until post-consent.

### Data collection

#### Shadowing and fieldnotes

Following the writing of a protocol and the publication of a protocol paper [33] preparing for this pilot study, the study researcher (EF-A), a post-doctoral research fellow with a background in sociology and extensive experience conducting qualitative research, conducted fifty hours of shadowing. EF-A followed members of the case team within and across workspaces. Each observation session was 5–7 h long and took place at different times of the day. To resonate with and understand WAD, shadowing included naturalistic observations [33] as people traversed surgical theatres a cafeteria and lunchroom, wards and corridors, and waiting and meeting rooms. Data quality was strengthened through rigorous recordings. Briefly written notes became more detailed, typed fieldnotes spatially contextualising events in a place-sensitive fashion [47]. The researcher was always cognizant of study aims and objectives during informal conversations, and at times steering the participant back to issues of relevance to the study, such as use of space, different use of devices and technology in spaces, team relationships in different parts of the OT or ward, etc. Such questions are not guided by a predetermined interview schedule nor were pre-prepared data capture tools such as case reports used. In terms of analysis, the conversational data is considered inseparable from the spatial, temporal, and social context in which it emerged.

#### Photographs, drawings and architectural plans

Photographs, drawings and architectural plans highlighted the layout of the workspaces, variations between space (number, shape, location, size, and open and closed areas), and the look of different spaces. Visual data supplemented fieldnotes and supported a multi-facetted understanding of areas covered. Drawings, diagrams and photographs (devoid of identifiable features or de-identified later) supplemented architectural plans and indicated spatial layout and function. These techniques, in line with teams’ prior research [2, 7] were designed to stimulate new knowledge of how people think, feel about their environment, and behave [48].

### Data analysis

Fieldnotes were analysed using thematic analysis with two experienced analysts (EF-A and FR) ensuring rigour and deriving consensus over issues of significance, categorised into common themes [49]. The secondary data analyst (FR) examined a sub-sample of data to ensure methodological validity, while the core study team met during the process of data collection to discuss the process and methods being used. Photographs, drawings, diagrams and architectural plans were subjected to their own discrete analysis through the team’s publicized schematic framework based on a visual taxonomy [2, 5, 7, 50]. The framework accounted for: content and context, object clustering and positioning, data ‘affect’ (feelings arising), frequency of spatial representation, and spatial function. The analysts met to discuss WAD during group-work meetings, comparing and contrasting images, while the analysis of architectural plans indicated the frequency distributions of the relationships between percentage spatial use and spatial arrangement. Visual and textual data were considered corroboratively. Data from EF-A’s fieldnotes were seen as of equal importance to visual data. Analytic frameworks were refined iteratively as data collection continued.

## Results

Six themes are reported (Table 1) comprising analysis of both fit- and unfit-for-purpose spaces. The quotations of participants were documented verbatim at the time of the event or shortly thereafter by the study researcher (EF-A) in written, de-identified fieldnotes. Pseudonyms have been included to protect identities and uphold confidentiality. Some diagrams, plans and photographs are included.

**Table 1:** Six Themes of WAD and Workspace Use in GI Surgery

Theme Number	Theme Title	Fit-for-Purpose Space or Unfit-for-Purpose Space
1.	Spatial Reminders Through Objects in Space	Fit-for-Purpose Space
2.	Accommodating Space	Fit-for-Purpose Space
3.	Sterile and Contaminated Space	Unfit-for-Purpose Space
4.	The Alcove	Unfit-for-Purpose Space
5.	The Changing Dynamics of Space	Unfit-for-Purpose Space
6.	Cold Space	Unfit-for-Purpose Space

### Fit-for-purpose space

#### Theme 1: spatial reminders through objects in space

Signs and objects around the hospital ward can act as a spatial reminder of where a HCP has to be at any given time, and what HCPs are expected to do in different parts of the hospital or on the GI surgical ward. This is particularly relevant and useful for surgeons, given that the demands on them are so varied.

**Fieldnote 1.1**

*We wait for the elevator to travel down to medical imaging. Arthur [surgeon] glances towards the doors to the recovery unit and the sign that sits above it and exclaims: “Oh!”. His finger points straight up and signals to the left and Arthur strides towards the recovery door. We enter and see a patient and his wife waiting patiently. Arthur apologies and they discuss the patient’s post-surgery home care … We arrive at medical imaging and check Pam’s [patient’s] scans. As we wait in the hallway, Arthur tells me that he’s been thinking about my interest in space and that he: “wouldn’t have remembered to go and see that patient in recovery, because although Eliza [scout nurse] asked me, no one wrote it down”. I explain the concept of a dialogical relationship to Arthur, and wonder if such a relationship can occur outside of a person-to-person interaction, can the space itself tell you what to do next? This idea seems to resonate with Arthur, and he agrees, “yes, I had forgotten, but as we were waiting by the elevator, I saw the doors to recovery and that reminded me … The space reminded me!”* (27 August 2018)

Space in this respect is more than an inanimate or taken-for-granted part of a professional’s work, it is an active ‘go-between’, helping maintain good relationships (Fieldnote 1.1).

Objects in spaces are also key to preparing staff for the role they are about to enact and provide key indicators to tasks about to take place so that staff can mentally prepare. The elevator to the medical imaging suite, the changing room where staff dress in scrubs ready for the day’s surgery, and the area directly next to, but not actually a part of, the operating space, for example, help staff move from one state of mind to another. As staff put on their scrubs or as the surgeon picks up a scalpel a quiet descends and sense of calm, enabling a different work experience to take root.

#### Theme 2: accommodating space

The hospital is a wealthy, private, metropolitan site where public and private areas are widely available to patients and their families. Fieldnotes 2.1–2.3 illustrate how both public and private spaces are accommodating spaces, where caring conversations between patients and HCPs are enacted, even in predominantly ‘open’ areas (Fieldnote 2.1).

**Fieldnote 2.1**

*We leave Theatre 21 and head towards the front desk where we run into Yvette [Arthur’s patient]. One of the nurses is gaining her written consent for the procedure. Arthur stops to introduce himself. She clings to the bedclothes pulling them closer to her body and appears slightly concerned. He asks, “Are you cold?” and she nods. Arthur disappears around the corner and retrieves a towel and white hospital blanket. He hands the towel to me and instructs me to hold onto it. He pulls down Yvette’s existing hospital blanket and folds the new one into a rectangle and lays it on top of her and then pulls up the existing blanket. He then takes the towel and wraps it around her head. She smiles and thanks him.* (21 June 2018)

In preoperative patient rooms, spaces are large, with attached bathrooms. Individual rooms mean space is flexible, accommodating not only HCP work, but also private and personal exchanges (Fieldnote 2.2).

**Fieldnote 2.2**

*We return to the wards but stop to visit Rachel. Her husband sits in the chair to her right. Arthur introduces me to her, and I take a seat and so does he. Both Rachel and her husband smile at me warmly. Rachel sits up in bed, less than a metre away from Arthur:*

*A: We’ve had a surgery run late, much later than we’d hoped. And because yours is an elective surgery I’ve come to talk to you about whether we should do it.*

*R: Oh no. But I’ve fasted and everything, and I know it’s elective but as soon as we said we’d do the surgery, I’ve been in so much pain. The last two weeks, I’ve had pain every day.*

*A: Hmm … . You see, my operating partner, Paul, who I usually do surgeries with has to go before your surgery, he actually has to get on a plane … he has to go, and so we can still do it but it won’t be as good.*

*R: Oh, I really don’t want to have to wait.*

*A: And I really wanted to come in here and for you to look at me and tell me, “Oh Arthur you look so tired”.*

*R: We did think this was a possibility, when we heard you were coming up on the ward to see us. [Rachel looks at husband who nods in agreement].*

*Husband: We should discuss it, but my immediate thoughts are, if we’re going to do it, we should do it under the best possible conditions.*

*A: I can leave you to discuss it?*

*R: When would the surgery happen?*

*A: In two weeks, maybe, but I can’t promise anything.*

*R: Two weeks! I can’t wait that long.*

*A: We’d make sure you were the first person on the list, so this doesn’t happen again.*

*A: You’re probably going to be a bit angry with me when you leave but we want it done right.*

*Rachel and her husband make plans with Arthur for the upcoming surgery and he tells the nurse not to charge them for the current stay. They part on good terms, having accepted the unwelcome news. We return to the operating theatre. As Arthur enters Theatre 21 he says, “well I’ve convinced Rachel to delay her surgery”. Adrian [scout nurse] stands in front of us and raises his hands into the air and yells “YES”. He is clearly one of many relieved staff members to hear this news.* (5 July 2018)

As Fieldnote 2.2 illustrates, accommodating space can engender negotiations between people that might not otherwise take place, as they bend or stand firm on a particular point. In the case of patients waiting for an important operation, accommodating space can help arrive at accommodating decisions. Occasionally, accommodating spaces even allow staff to exchange confidences and personal exchanges (Fieldnote 2.3). ensuring a sense of sanctity within which to enjoy a quiet moment.

**Fieldnote 2.3**

*On Monday afternoon, as I stand behind the equipment next to Arthur who is furiously writing up his notes, I catch two of the nurses (one who has participated in the operation and one who has not) who stop to chat. One comments on the anaesthetic nurses’ lip-gloss and she produces a small container of it, explaining that it is new. She removes the cap to reveal a pink hue. She offers it to the other nurse to smell, who exclaims with delight at the scent as if to affirm the well-selected new purchase. I note down the interaction in my book and Arthur momentarily looks up and asks me what is happening. I explain and he smiles and returns to his notes.* (14 June 2018)

In all examples, accommodating space allows for exchanges to be varied and run their course, often moving from a purely professional, to a private footing. Accommodating space enables patients to approve but also show dissatisfaction with, professional activity (Fieldnote 2.2). It encourages them to voice their pain, disappointment, or hopes for the future (Fieldnotes 2.1 and 2.2) while ‘nice’ or well-appointed spaces serve to set the tone, style, and level of conversations (in ‘nice’ spaces discussions are polite and respectful).

### Unfit-for-purpose space

#### Theme 3: sterile and contaminated space

Surgical spaces are demarcated as either sterile or contaminated areas (Fieldnotes 3.1, 3.2, 3.3 and 3.4). Unit members belong to one or the other, indicated, more or less obviously, through their bodies, colour coding, and the clinical devices they use, such as their surgical instruments. All staff members monitor the boundaries between these spaces through their bodies and devices, so that transgressions cannot occur. Staff members also demonstrate an awareness of the sterile and contaminated spaces in the way they performed their duties, and in their ability to contain their own body movements to the appropriate space. Moreover, staff manage garments or instruments to make sure they do not transgress boundaries. Keeping instruments close to hand at all times, means one can ‘claim’ them for oneself.

**Fig. 1:**
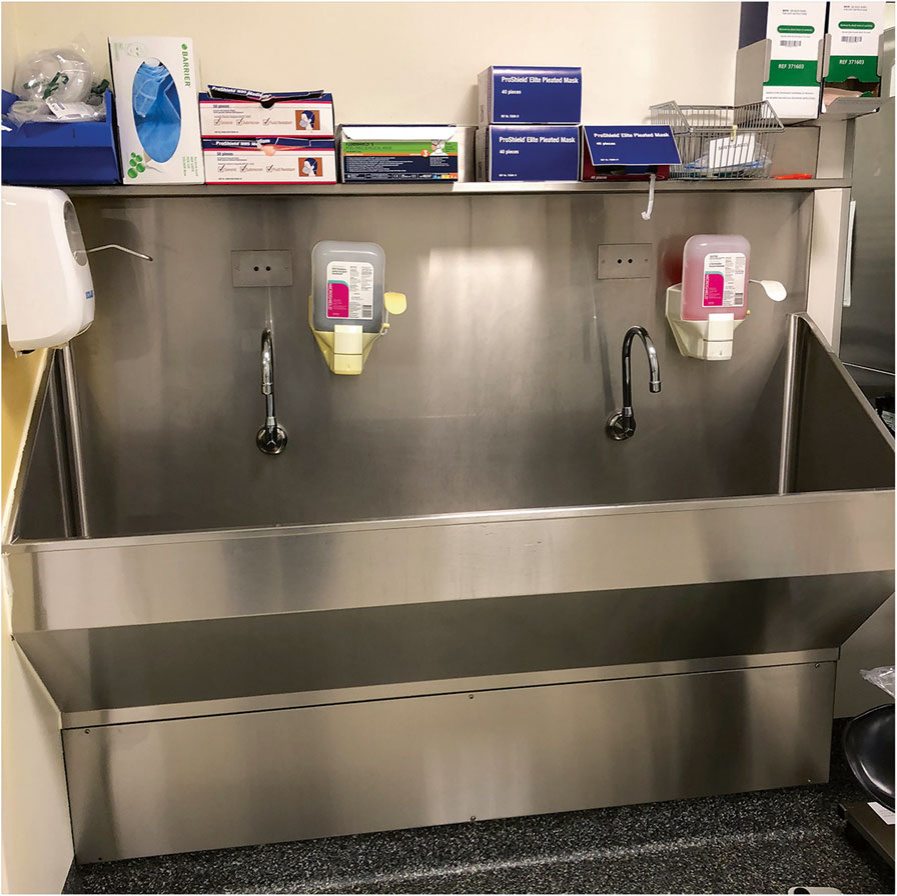
Sink where surgeons ‘scrub up’ outside Theatre 21

Maintaining boundaries of pre-demarcated, sterile space is important for maintaining patients’ health and safety (Fieldnote 3.4 and Fig. 1) and the management of the team’s physical proximity or distance from one another.

**Fieldnote 3.1**

*The washing process takes 5 minutes or so … It seems excessive, but I have read about a surgeon’s elaborate washing rituals before … When he sees me watching him, Arthur comments: “You have to get into all these places you wouldn’t usually wash”. After cleaning his nails, he spreads out both set of fingers and using the other hand, scrubs between each finger. The sink is large enough to accommodate such a process, it is broad and deep, and the shelves do not jut out, so Arthur has the height to move his arms up and down and from side to side.* (27 August 2018)

**Fieldnote 3.2**

*Arthur exits from scrubbing up, uses his foot to push open the door... and strides across the room to retrieve a small blue cloth from a silver table to the left of the anaesthetic room doors. His steps are purposeful and fast-paced and command a sense of authority. He spends a good half a minute drying his hands, wiping in between his fingers. He holds the top end of the gown and with one firm stroke, the gown unravels quickly. Although I have seen Arthur do this several times before, this always appears dramatic and theatrical... Only Arthur inhabits this space, except for the moment when he has his gloves on and needs a nurse to fix the gown at the back of the neck and middle of his back.* (5 July 2018)

**Fieldnote 3.3**

*Jenny [scout nurse] has a long-sleeved, navy blouse over her scrubs which is tied at the waist. I look around and realise that all the (female) nurses are wearing their uniform this way. I ask Jenny why and she tells me it’s so her top does not touch the sterile areas. To make her point, she undoes the tie and the shirt flaps around loosely. She adds, “the buttons also don’t really do up, they always pop open”. I take a closer look, and they are indeed the feeble silver clip type which seem to be of poor quality. She undoes a few and tries to do them up, surprised by the few that stay fastened.* (5 July 2018)

**Fieldnote 3.4**

*David, Matilda and Madeline [team members] stand over near the entrance doorway in a semi-circle; they look at ease... They wear their blue coats loosely … they have no reason to go near the patient’s body, maintaining sterility of the space is not their priority. In contrast, the scout and surgical nurses are constantly dealing with issue of sterility. The scout nurses live on the boundary between the sterile and contaminated areas and their actions, and use of space, reflect this fine line.* (12 July 2018)

While sterile and contaminated spaces are clearly predefined, members of the team constantly make autonomous decisions about the way these spaces should be managed. This includes how to wash, what to wear, how to avoid certain areas and how to uphold sterile practices. Staff also decide which actions to avoid and when greater flexibility, in terms of relaxing boundaries or maintaining sterile spaces, is needed. How people act and view these changes enables a team to continue to adapt to, or control space in a way that suits them best. However, if this is out of kilter with the changing needs of a unit, such as when there is the need to reconfigure space slightly to suit different operations, tensions can rise. There is a fine line between what constitutes suitable and unsuitable spaces and when that line is overstepped, harmonious working relations can break down.

#### Theme 4: the alcove

**Fig. 2:**
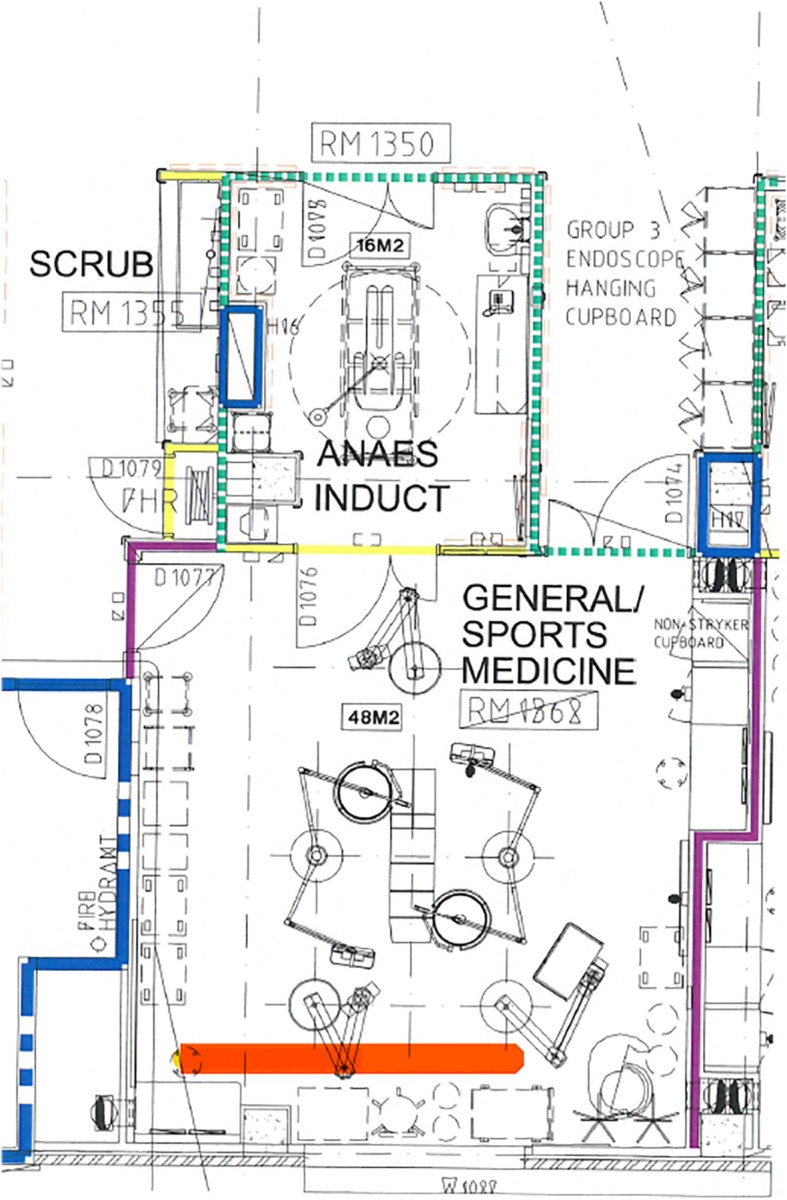
Architectural plan of Theatre 21

**Fig. 3:**
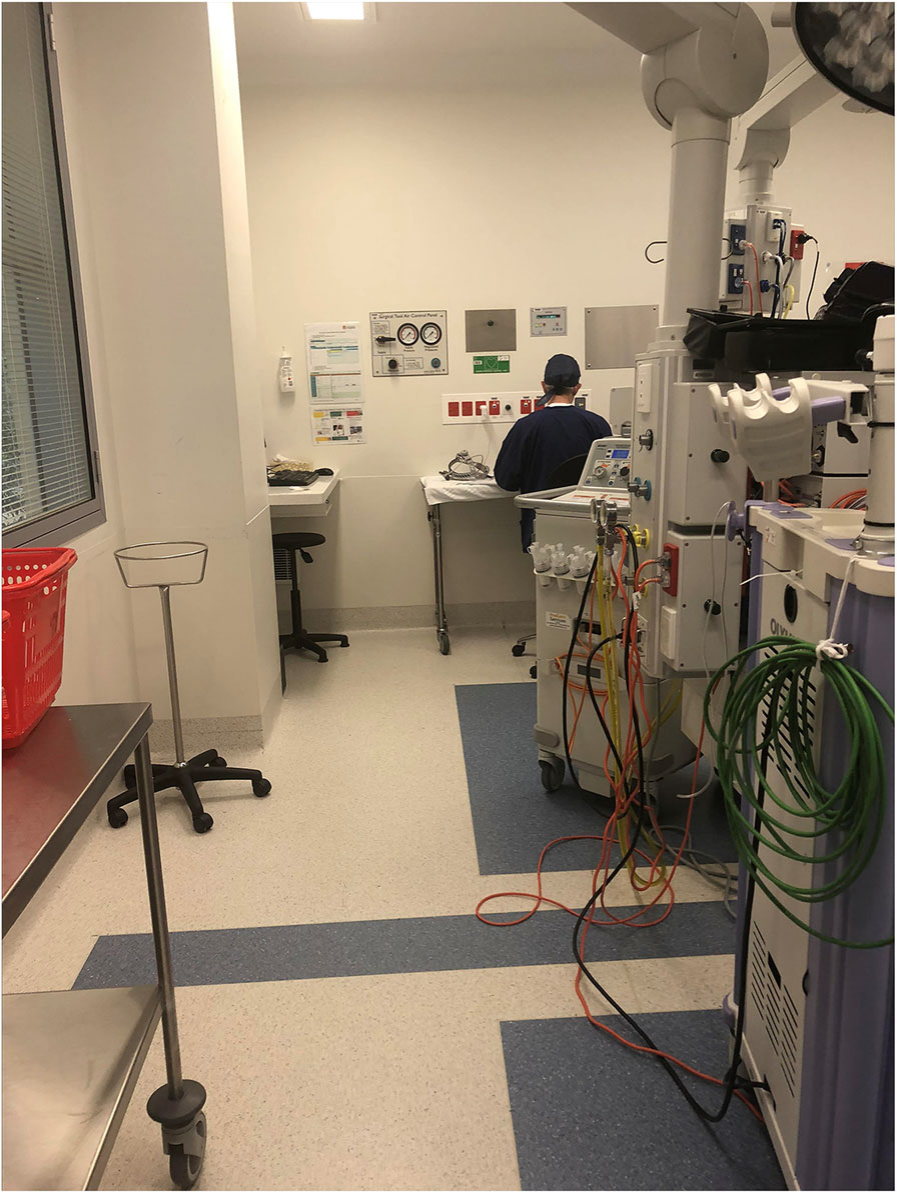
Arthur writing notes in the Alcove

The surgical theatre contained a narrow, small alcove at the back of the room where Arthur had a stool and a makeshift desk (actually an instrument trolley) to support his laptop (Orange highlight in Fig. 2). After surgery, he sat there quietly to write up his surgical notes, communicate with others, and complete a range of administrative tasks (Fig. 3). Prior to surgery, Arthur used this alcove to review patient notes and talk to his surgical partner (Fieldnote 4.1). The structure, size and location of the alcove afforded Arthur some, yet not complete, relief from staff interruptions (Fieldnote 4.2).

**Fieldnote 4.1**

*Arthur takes a quick phone call between surgeries about a topic unrelated to the operation. This is conducted in the alcove, where his two computers reside, and the monitors mostly shield him from the view of others. It acts as a makeshift office within the theatre. Although he is generally a chatty, vibrant man, it is an unspoken rule that he is not to be disturbed during his writing time. In this space Arthur is bounded by quietude, his back facing the entrance.* (14 June 2018)

**Fieldnote 4.2**

*I stand next to Arthur and I am careful not to say anything, I know he is busy writing up his notes. Amalia [surgical nurse] comes to talk to him and asks Arthur to sign a medical certificate to acknowledge the completion of the surgery. Five minutes later, Sophie [trainee scout nurse] comes to tell him that he needs to see Mr Allen [patient] on the ward. After she leaves, Arthur says: “It’s worth noting that … The constant interruptions, it’s irritating, but they won’t see that.”* (5 July 2018)

Bounded space has a vital role to play, not only in maintaining sterility, but also, as in the example of the alcove, in the personalisation and demarcation of space. While bounded space is rare, when in evidence, it is also useful for establishing hierarchy. Within the alcove, the surgeon is ‘king’, creating a recognised distance between himself and his team, and while indicating who is in charge and what role divisions entail, bounded space upholds the need for regulation. However, without a physical barrier, there is always the opportunity that boundaries will be breached (Fieldnote 4.2). The distance between people and spaces is, therefore, forever being tested, often foreshortened, and even here, interruptions can occur.

#### Theme 5: the changing dynamics of space

During surgery, staff members beyond the core surgery team frequently enter the ward’s theatre through the exit door, to seek advice, gather information, or for purely social reasons. In most cases, additional personnel are a temporary interruption. However, during challenging surgeries space can become overcrowded and noisy. This sometimes leads core team members to become distracted and if requests for assistance cannot be heard, to raised voices, to ask for quiet in which to concentrate.

When distractions occur, reprimands follow and the dynamics of space change (Fieldnotes 5.1 and 5.2). Dynamics can also change when surgery is conducted by pairs of surgeons. Then surgeons become removed from other staff members, talking together and depending on each other (Fieldnote 5.1).

**Fieldnote 5.1**

*Arthur and Paul [paired surgeons] talk to each other constantly throughout the surgery, but it is difficult to hear what they’re saying. They only raise their voices when a request is made to another staff member. Although we are all in the same room, the dynamics of the team is different today. There is an absence of social conversation and Paul and Arthur talk primarily to one another, except for when they require something.* (5 July 2018)

**Fieldnote 5.2**

*David receives a work-related phone call and after thirty seconds or so, Arthur interrupts him and requests that he moves outside. Sophie and Louise [scout nurses] sit next to one another in the corner to chat, and Louise points to the computer a few times. Arthur stands parallel to the patient, watching the monitor, and Paul stands in between her legs. In a loud voice, Arthur says: “I don’t know what you’re talking about, but can you guys stop talking over there?” Offering an explanation, Paul adds: “It’s turned into an intense surgery”. Five minutes pass and Sophie and Louise begin to chat again in low voices. Arthur responses almost immediately, “I know I’m sounding like a real ___ and I know you’re whispering but I need to NOT see you guys talking out of the corner of my eye.”* (5 July 2018)

**Fieldnote 5.3**

*Arthur no longer looks bright and energetic, and he walks as fast as usual, but I suspect not in haste to get to his next patient. He tells me that we are not going to the lunchroom to eat today (situated next to the surgical theatres), he needs something more “substantial”. We head to the downstairs cafeteria where we can talk freely. After twenty minutes of chatting, Arthur tells me that his internal alarm clock is going off and that we must return to the theatre. I have not finished my lunch, so I quickly eat the remainder of my sandwich and take a last mouthful of hot chocolate.* (5 July 2018)

Within an ever-changing dynamic, different groups of people conceptualise space in different ways, from those with hierarchical presence, to those temporarily entering and exiting the theatre [51]. The more people that use a space, the greater likelihood that disruptions will occur, leading to disjuncture in working habits and work relationships and the potential for mistakes being made. When two surgeons work together, or when one surgeon experiences the intensity of an operation, the stakes are higher. Then space closes in and becomes even more regulated. For the individual surgeon, a large space can become a distraction. In these instances, space is less casual and more intense. We see examples of this in the fieldnotes, when at times the surgeon needs to exert his authority. Yet spatial-use and team expectations alter so rapidly that people behave in unexpected ways, wielding power, or testing the boundaries, with unpredictable consequences.

When surgery is over, and one of the surgeons and the researcher meet to relax, space expands (Fieldnote 5.3). Then, two different individuals can share a less formal conversation, enabled by the particularity of the context. Beyond the intensity of the theatre and the privacy of the patient’s bedroom is the laxity of the cafeteria. This space, peripheral to team working, is at the *Edgelands* of what might be seen to be the case workspace [52, 53]. The dynamics at the Edgelands always affords certain freedoms. This is often the case in unregulated spaces inhabited infrequently. Here, two individuals can make different kinds of choices about how to act. At the Edgelands, for a brief moment in time, two individuals can converse on a more equal footing, dipping in and out of different kinds of conversations.

#### Theme 6: cold space

**Fig. 4:**
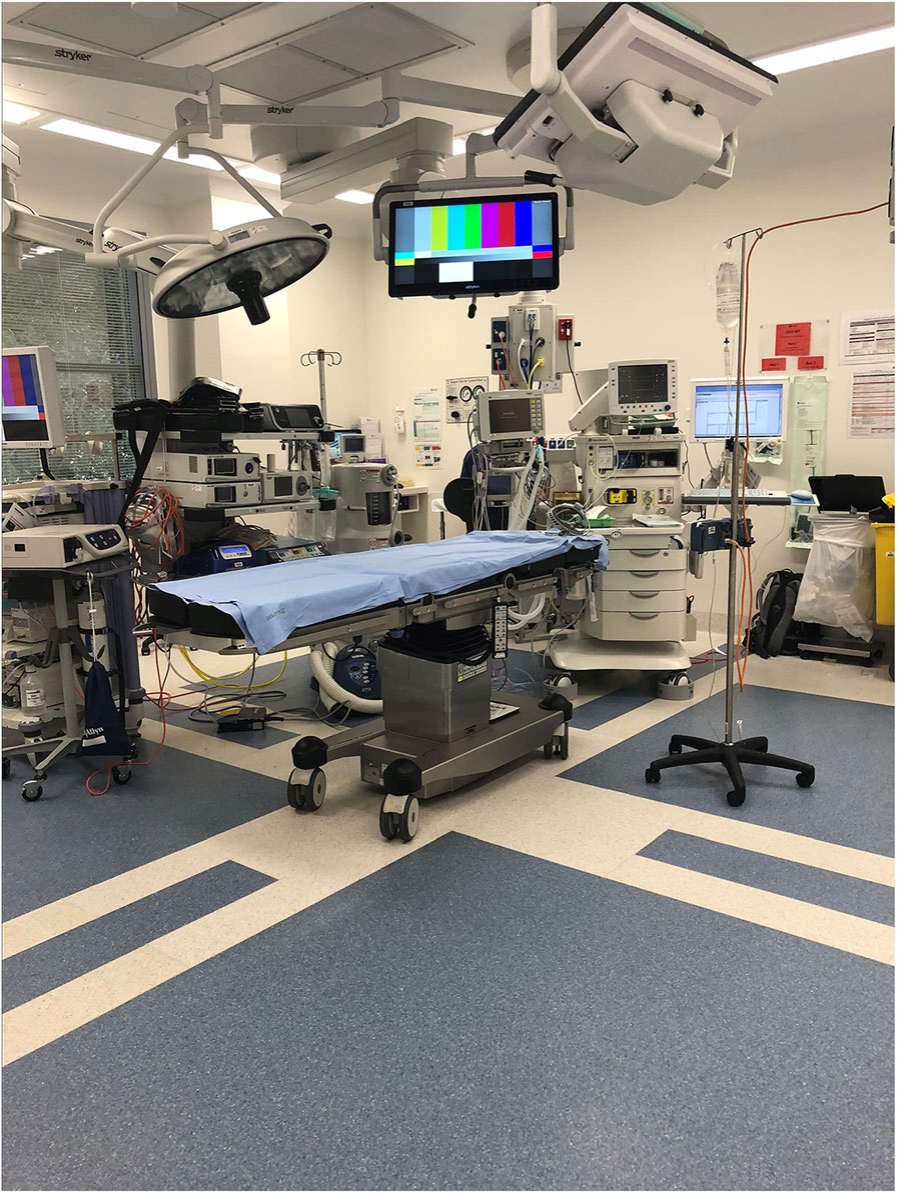
Airconditioning vents directly above where surgeons stand in Theatre 21

The surgical theatre was a cold space in physical terms. The temperature was kept at a minimum, to adhere to the regulations of surgical procedure laid down by the hospital and enhance the sterile environment. However, the cold space appeared to affect most staff members and even some patients, with the placement of an air-conditioning unit directly above the surgical table, creating particular difficulties for Arthur as he operated (see Fig. 4). Arthur often wore small towels or pieces of cloth around his neck to keep himself warm, and would, on occasion, move the overhanging lights so that they sat directly between his head and the air conditioning unit and thus acted as a shield against the cold air (Fieldnote 6.1 and Fig. 4).

**Fieldnote 6.1**

*As Arthur performs the colonoscopy, he returns to his discussion about motorbikes, and tells us that he once owned a Yamaha 650: “I still have dreams about it, it was smooth”. Arthur shivers and looks up … in an irritated tone he says, “I’m right under the … ” He holds the colonoscope with one hand and reaches up with his other to pull across the large light which now shelters him from the cold air pouring out of the air-conditioning directly above him.* (27 August 2018)

In this theme space is not always welcoming and supportive. Indeed, space can be seen to be an obstacle and an intrusion, with disabling features and qualities. Then space works against effective team functioning, creating a barrier between people and productive working, and instilling tensions in the team at critical moments. When space is intrusive, its qualities cannot be ignored. This depends on the balance between the intrusion and the ongoing functionality, but when space is unsupportive the team often looks for creative ways to ensure its effective management, relying on ‘workarounds’ (Fieldnote 6.1) to overcome problems and to ensure people can continue to work alongside one another harmoniously.

## Discussion

This proof-of-concept study has generated in-depth knowledge of one GI surgery unit’s use of workspace and has created an evidence-base for understanding WAD (rather than WAI) in a modern-day, metropolitan, Australian hospital. Rich detail was disclosed through mobile methods supported by strong collaboration between researchers and clinical staff. Findings have the potential to be tested in other studies and sites across Australia and the methodology expanded to elsewhere. The six study themes act together to illustrate key aspects of workplace design that need to be considered to ensure fit-for-purpose spatial requirements across systems, with flexibility built in to optimize people’s health, wellbeing and productivity as well as their safety.

In the discussion, we examine the need for flexible and accommodating spaces that are expansive yet bounded. We consider the importance of the spatial *aide memoire* and the physical comfort of the team, and examine the need for team members to stop having to search for ingenious ways to circumvent unfit-for-purpose spaces.

### Flexible and accommodating spaces

From the field of human factors research, we know that poorly designed workspaces constrain the fluid enactment of work (whether in healthcare or other industries [54]). Flexible workspaces, on the other hand, have proven effectiveness for different workforces, such as the ‘knowledge worker’ (whose job involves handling and managing information [55]) and emergency department and critical care unit staff [56, 57]. Flexible workspaces can support improved communication, teamwork and security, while accommodating space is noted as being more pleasant to work in [58]. Thus we agree with Dean and colleagues [59] that an increasing reliance on teamwork requires new flexible configurations for shared decision-making, patient empathy and sensitive negotiations with patients to be enabled during emotive encounters.

### Clear, non-negotiable boundaries

We also concord with others’ conclusions [60] that invisible, yet non-negotiable boundaries are effective ways of mapping certain spaces. In the theatre, for example, they demarcate sterile from contaminated areas, and maintain and uphold important rituals [60] such as a team’s pre-operative washing and dressing routine. This helps establish surgical spaces that are discontinuous from their surroundings, and thus contributes significantly to a particular ‘mind set’ [36]. We discovered that the act of handwashing is not only a necessary ritual of cleanliness, but also an enabler for the experienced surgeon to ‘set’ their mind to the work about to commence; in effect to transmute into a calmer persona.

We found the division between sterile and contaminated areas was also a proxy for other boundaries. For example, surgical versus non-surgical spaces reflected ‘core’ or ‘wider’ team domains, while divisions become a way of controlling group relations (who is privileged to inhabit this space and who is not). In this respect, porters and other HCPs were not always welcomed into the privileged, sterile theatre, and could be more easily singled out as, ‘outside the inner circle’ – peripheral to the team’s spatial imposition. Manipulating space in this way is not, however, unique to healthcare. In aviation, there is a strong demarcation between the flight deck and the aircraft cabin, where rules about who can enter the flight deck and when, serve to maintain control and status [61, 62].

Sterility and hygiene can only be accomplished through the collective actions of a team and must be continually addressed [35]. While no individual team member can ensure that boundaries are maintained, despite colour demarcation, together, teams can carefully monitor their movement, clothing and instrument-use, to avoid transgressions. Given the vital importance of preventing infection, control over oneself, others and objects has been described as an embodied “hygiene capital” [63].

In addition to clear, non-negotiable divisions between sterile and non-sterile spaces, bounded space is important in other, artificially-imposed ways (not necessarily part of an original design). Bounded space can help meet the needs of individuals and teams (seen in the example of the alcove), whereby having a space to oneself increases feelings of commitment to an organisation [64]. Nevertheless, claiming space without negotiation can be unnecessarily territorial [64]. This suggests that for teams to function effectively, creating a range of bounded spaces with opportunities for expansion, where people can express their individuality is beneficial as it alleviates the ‘divide and rule’ mentality. This can also extend to the idea that space has been purloined for another’s use. The literature indicates that small ‘offstage’ spaces can be personalised to provide a degree of privacy and offer a temporary disconnection from busy surroundings, and help mitigate against fatigue in the workplace [65], but without the provision of such spaces, individuals may give the impression that they are detached or disinterested, with implications for both interprofessional relationships and patient care experiences [66].

### Expansive space

The changing dynamics of space, as members of the team move through the hospital, influences how people work and communicate. ‘Shared’ spaces such as corridors, that are not traditionally ‘owned’ or occupied by any one group can act to mitigate normal hospital hierarchies [67] or constraints on team members. This enables greater variation in WAD and opportunities for openness with patients and others. Variation in individual behaviour in the common spaces also results in less predictable responses to encounters between staff members, and staff with researchers and patients. While there are potential benefits to closer interaction, such as a greater sense of connectivity [68], there are also associated distractions and disruptions, giving rise to possible misunderstandings or conflicts. Thus, there is also the need for some boundaries to be dissolved or adapted for a new personal dynamic to evolve, heralding greater inclusivity and sympathy.

### The aide memoire

The importance of objects in spaces, as cognitive aids, is under-estimated in the healthcare literature [69]. Our study indicates that objects are vital prompts in reminding people to complete a task, and that the *aide memoire* should be more successfully, albeit discretely, integrated into the healthcare context. Research evidence for the usefulness of cognitive aids to help with the completion of tasks is, however, mixed [70] and while the *aide memoire* has been used in surgical record keeping [71, 72], its value has up to now been mostly limited to written texts derived from guidelines. This study indicates the value of expanding our investigation into how people use the *aide memoire*, looking more closely at how people use physical and spatial objects as memory cues, and their value for grounding clinicians preparing for the next task. New research could also examine how to leverage design to improve cognition and performance.

### Physical requirements of a team

In the context of WAD, the clinical team have clear physical requirements. They need, for example, an environment that supports them as individuals but also one that is suited to the job in hand. Surgical environments, we are told, must include cool temperatures and sufficient airflow for surgical sterility and good air quality. But while the literature tackles this in terms of surgical infection [73], it does not dwell on the physical needs of the team, whose comfort can run counter to this, creating tension. While guidelines for operating theatres indicate that: “the temperature of ORs [operating ranges] should be kept between 68 F (20 C) and 75 F (24 C)” [73], in this study one surgeon was sufficiently discomforted that he had to find creative ‘workarounds’ like wearing more clothes. Indeed, over time, working in an uncomfortable environment can generate stress as energy saps and people are directed away from their work to new coping strategies for adverse conditions [74]. We need to factor in comfortable working environments, including adjustments to the placement of air-conditioning units and suitably protective clothing.

### Circumventing unfit-for-purpose spaces

In this study surgical theatres and clinical and non-clinical workspaces are highly illustrative of the management of WAD. People can be negatively affected by unfit-for-purpose spaces, as the case team indicates, looking for ways to circumvent problems and spending time and ingenuity delivering creative spatial options, whereby alcoves become offices and theatres become walkways. This not only eases patient throughput, it also helps with more general professional access, so people can function effectively while moving from one space to another. While new habits clearly help staff manage the negative implications of unfit-for-purpose spaces, they can also lead to unexpected consequences. This includes staff regularly ‘rewriting the rules’, talking during difficult operations, crossing sterile boundaries, and sharing confidences in surprising ways. Staff can go ‘rogue’ – reinventing themselves and their spatial layout and adapting clothing for more sustained precision medicine practices so work demands can be met. However, it is important to note that ongoing adaptation needs to be coupled with ongoing awareness of the safety implications of ‘workarounds’, for sustained, effective performance.

## The value and implications of mobile methods in other medical settings

As the first Australian case study of its kind, mobile methods have been uniquely tailored to investigate a GI surgery unit’s use of hospital workspace. In-depth, nuanced data clarified spatial design and organisational productivity, while knowledge transfer between researchers, HCPs and hospital managers led to a clearer understanding of the strengths and weaknesses of different spatial arrangements from the perspective of healthcare staff. Insights indicated new spatial improvements would benefit all concerned. This study has the potential to be expanded to other units at the study site, and other settings and wards in and beyond the hospital in question. Having undertaken this pilot study, we now plan to implement mobile methods in a larger study examining surgical theatres and wards across both private and public hospitals in Australia. Transferring methods to other sites and settings will allow the study team to test the mobile methods methodology in other contexts for its adaptability and rigour.

Mobile methods have substantial value in supporting investigations into real-time WAD in healthcare contexts, with the potential for outcomes to inform more resilient healthcare [75, 76]. Internationally, this field is in its infancy in terms of qualitative methodological developments that are suitable for assessing WAD from the perspective of clinicians delivering patient care. We believe these methods can be adapted across healthcare contexts and settings, and could be applied across national health systems globally. Their use could be expanded to examine, for example, safety culture, integration of devices and technologies in workspaces, and clinician teams and teamworking, However, it is important to emphasise that mobile methods’ data capture cannot be managed using standard, survey-type documents, case reports or structured interviews. Rather, it is vital that researchers observe in-situ behaviours and activities discretely, taking fieldnotes at appropriate times and being fully aware of informal conversations taking place. Furthermore, the methods demand researchers who can adapt quickly to circumstantial changes, adjusting, for example, to the practices and behaviours of those around them. Researchers can be trained in mobile methods (both the theory and the practice), learning from others through shadowing and mentoring or through taught Master Classes and once familiar, whilst differing from static interviews and focus groups, the methods will add exponentially to their skill-mix. Expertise in this methodology will not only add to an individual researcher’s choices of methods for research conduct but also equip them with techniques to deliver insightful research findings and understand behaviour-change and system adjustments amidst healthcare and other services undergoing accelerated rates of change.

We have evidenced how mobile methods can enable rich visual and textual data and contribute to our understanding of how small and large adjustments to spatial arrangements successfully achieve desired goals. This offers a baseline from which to develop further research into WAD, applying knowledge to a larger cohort of participants serving a wider population, to produce a set of quality improvement measures for hospital design. Not only could this optimise healthcare workspaces, but ensure more productive, empathic working practices that reduce workforce stressors, enhance physical and psychosocial wellbeing and afford patients and professionals safer environments in which to share decisions.

## Strengths and limitations

This was the first study of its kind to be undertaken in GI surgery in an Australian hospital context. Employing mobile methods enabled a dedicated study researcher to get close to a specialist GI surgery unit and investigate work regimes and relationships. However, the sample size (one small, fixed team, in a metropolitan setting) and the site location (one private, affluent hospital serving a homogenous population group) limits the scope of the findings and the transferability of messages to other sites and settings.

It is also possible that the presence of the researcher and the participants’ knowledge of the study influenced their behaviour to some extent, but to limit this, the researcher spent an extended period of time with the participants on each occasion and was present and at different times of the day to encourage naturalistic behaviour from all parties.

## Conclusion

In clarifying the role of workspace design in the everyday working lives of a GI surgery unit this study has revealed how seemingly inconsequential design choices can significantly affect the well-being of team members and potentially impact their performance. Furthermore, the study has illustrated that if we do not design workspaces to meet people’s needs, workarounds will be inevitable with unexpected consequences. While workarounds may be successful in problem-solving in the short term, they can lead to longer-term complications, such as less accountability and a lowering of standards.

By designing safe and harmonious work environments that support team communication and movement, we could build safer environments within which teams learn to function. Arrangements of workspaces, and their effect on happiness, health and productivity, as well as their impact on safety, disclose important considerations for workspace design. We recommend the construction of adaptive and protective workspaces and greater opportunity for promoting accommodating, dynamic spaces to engender positive conversations and outcomes, alongside greater adaptability in the design of hospital devices, objects and workforce attire.

Mobile methods have proven invaluable in this context and should be introduced more widely as a tool for investigating WAD. The methodology offers a clear understanding of what workforces are actually achieving in the hospital ecosystem, while enabling the formulation of recommendations for hospital design that could transform hospitals into better-quality, safer places. In such workplaces, teams will have the opportunity to work more harmoniously, happier in the knowledge that they are functioning in a fit-for-purpose environment.

## Data Availability

The data that support the findings of this study are available on request from the corresponding author FR. The data are not publicly available as they contain information that could compromise research participant privacy/consent.

## Abbreviations

GI: Gastroenterological
HCP: Healthcare professional
WAD: Work-as-Done
WAI: Work-as-Imagined
